# A Reference Genome from the Symbiotic Hydrozoan, *Hydra viridissima*

**DOI:** 10.1534/g3.120.401411

**Published:** 2020-09-08

**Authors:** Mayuko Hamada, Noriyuki Satoh, Konstantin Khalturin

**Affiliations:** * Marine Genomics Unit, Okinawa Institute of Science and Technology Graduate University, Onna, Okinawa 904-0495, Japan; † Ushimado Marine Institute, Okayama University, Setouchi, Okayama 701-4303, Japan; ‡ Zoological Institute, Kiel University, Kiel 24118, Germany

**Keywords:** green hydra, *Hydra viridissima* A99, whole genome sequencing, *de novo* assembly, symbiosis

## Abstract

Various *Hydra* species have been employed as model organisms since the 18^th^ century. Introduction of transgenic and knock-down technologies made them ideal experimental systems for studying cellular and molecular mechanisms involved in regeneration, body-axis formation, senescence, symbiosis, and holobiosis. In order to provide an important reference for genetic studies, the *Hydra magnipapillata* genome (species name has been changed to *H. vulgaris*) was sequenced a decade ago (Chapman *et al.*, 2010) and the updated genome assembly, Hydra 2.0, was made available by the National Human Genome Research Institute in 2017. While *H. vulgaris* belongs to the non-symbiotic brown hydra lineage, the green hydra, *Hydra viridissima*, harbors algal symbionts and belongs to an early diverging clade that separated from the common ancestor of brown and green hydra lineages at least 100 million years ago (Schwentner and Bosch 2015; Khalturin *et al.*, 2019). While interspecific interactions between *H. viridissima* and endosymbiotic unicellular green algae of the genus *Chlorella* have been a subject of interest for decades, genomic information about green hydras was nonexistent. Here we report a draft 280-Mbp genome assembly for *Hydra viridissima* strain A99, with a scaffold N50 of 1.1 Mbp. The *H. viridissima* genome contains an estimated 21,476 protein-coding genes. Comparative analysis of Pfam domains and orthologous proteins highlights characteristic features of *H. viridissima*, such as diversification of innate immunity genes that are important for host-symbiont interactions. Thus, the *H. viridissima* assembly provides an important hydrozoan genome reference that will facilitate symbiosis research and better comparisons of metazoan genome architectures.

The Cnidaria is an evolutionarily ancient and well-defined phylum, characterized by the possession of nematocytes (Brusca *et al.* 2016). Cnidarian species belong to the Medusozoa, which comprises the Hydrozoa, the Scyphozoa, the Cubozoa, and the Anthozoa (Figure 1A). Although cnidarian morphology exhibits astonishingly diverse forms and life styles, those of fresh water hydrozoans of the genus *Hydra* are relatively simple. *Hydra* possess only a polyp stage, while other medusozoans exhibit alternation of polyp and medusa stages.

**Figure 1:**
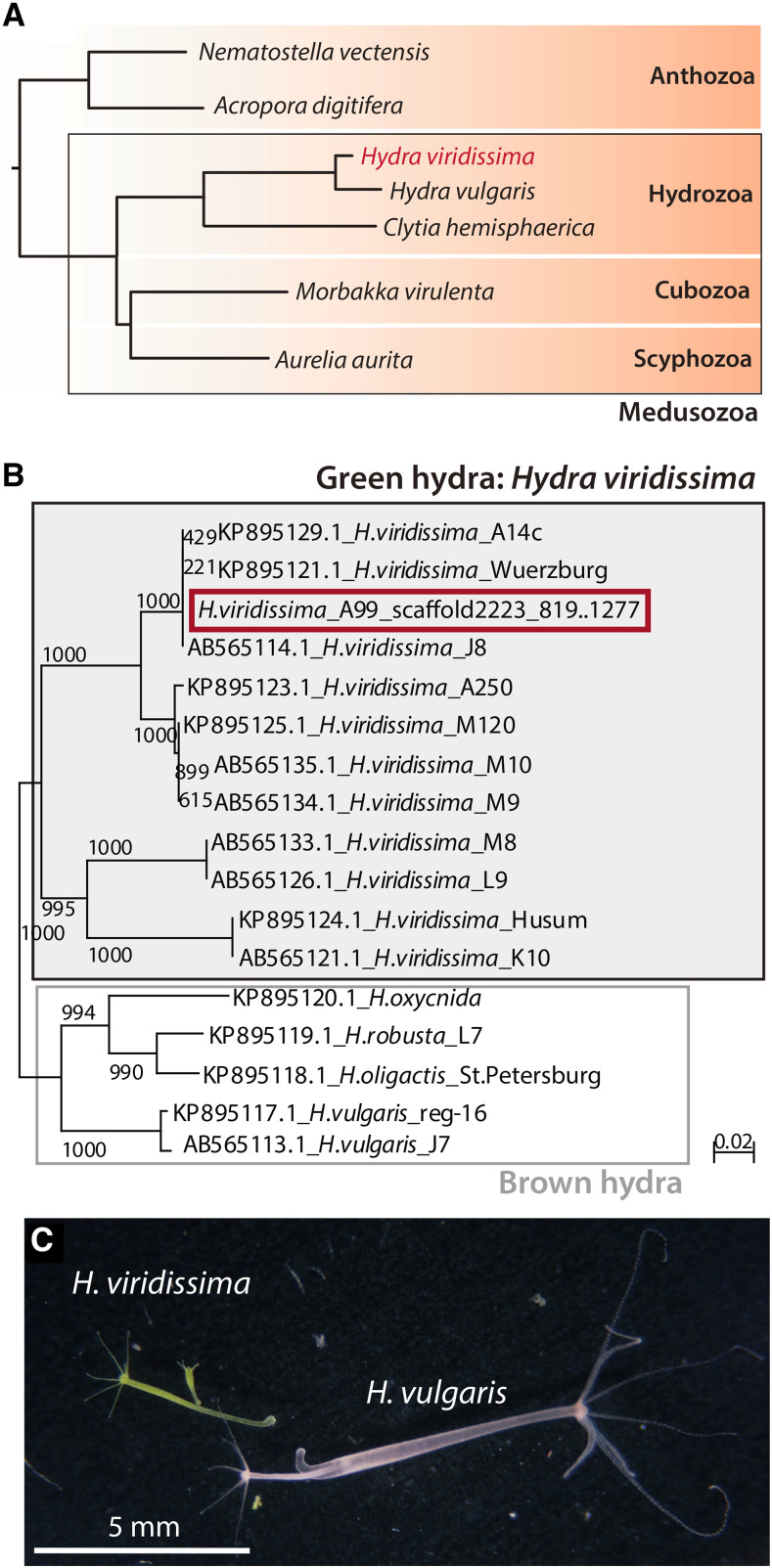
Phylogeny and morphology of green hydra *Hydra viridissima*. (A) Phylogenetic position of *H. viridissima* (red) within the phylum Cnidaria. (B) Relationship of *Hydra viridissima* strain A99 (red) with other *H. viridissima* strains and brown hydra species, based on phylogenetic analysis with the NJ method using cytochrome c oxidase subunit I (COI) gene sequences. The genomic region in *H. viridissima* A99 and Genbank IDs in other strains used in the phylogenetic analysis are indicated. (c) Photographs of *H. viridissima* (left) and *H. vulgaris* (right). *H. viridissima* is smaller than *H. vulgaris*, and green due to symbiotic *Chlorella* in its endodermal epithelial cells.

With its simple body structure and easy laboratory cultivation, *Hydra* has been an experimental model for studying cellular and molecular mechanisms underlying the formation of the body axis (Bode 2011), regeneration (Trembley *et al.* 1744; Bode 2003; Holstein *et al.* 2003), and also holobiotic relationships with microbiota (Deines and Bosch 2016). Introduction of transgenic and knock-down technologies further promoted these studies (Wittlieb *et al.* 2006). In order to provide genetic information for these studies, the *Hydra magnipapillata* (now classified as *H. vulgaris*) genome was sequenced in 2010 (Chapman *et al.* 2010), and an improved version was published in 2017 (Hydra 2.0 Web Portal: https://research.nhgri.nih.gov/hydra/).

While *H. vulgaris* belongs to the non-symbiotic brown hydra lineage, the green hydra, *Hydra viridissima*, establishes a mutualistic relationship with microalgae and exchanges metabolites with its symbionts (Figure 1C) (Muscatine 1965; Cernichiari *et al.* 1969; Mews 1980; McAuley 1991). While symbiosis with dinoflagellates is observed in many marine cnidarians, such as corals, jellyfish, and sea anemones, *H. viridissima* harbors the green alga, *Chlorella* (Douglas and Huss 1986; Huss *et al.* 1993/1994; Davy *et al.* 2012). According to several phylogenetic reconstructions, *Hydra viridissima* belongs to the basally branching lineage in the genus *Hydra* (Martínez *et al.* 2010; Schwentner and Bosch 2015) and its genome is much smaller than those of brown hydra species (Zacharias *et al.* 2004). Although all hydra species have a similar body plan, green and brown hydras are evolutionarily distant, and little is known about the genetics that enable green hydras to support this unique symbiosis with *Chlorella*.

Recent advances in genome sequencing have facilitated comparative analyses of cnidarian genomes. In addition to *H. vulgaris* (*H. magnipapillata*), genomes of representative species in each subgroup of cnidarians have been sequenced, including another hydrozoan, *Clytia hemisphaerica* (Leclère *et al.* 2019), the scyphozoan jellyfishes, *Aurelia aurita* (Khalturin *et al.* 2019; Gold *et al.* 2019) and *Nemopilema nomurai* (Kim *et al.* 2019), the cubozoan box jellyfish, *Morbakka virulenta* (Khalturin *et al.* 2019), the anthozoan sea anemones, *Nematostella vectensis* (Putnam *et al.* 2007), *Aiptasia* (Baumgarten *et al.* 2015), and various coral species, including *Acropora digitifera* (Shinzato *et al.* 2011). Here we report a draft assembly of the ∼284-Mbp genome of *Hydra viridissima* strain A99 as another high-quality *Hydra* reference genome. We report significant characteristics of the green hydra genome, including transposable elements, innate immunity-related genes, and genes that determine its body plan.

## Materials and Methods

### Hydra and extraction of DNA

The Australian *Hydra viridissima* strain A99, which was kindly provided by Dr. Richard Campbell, at the University of California at Irvine, was used in this study. Polyps were maintained at 18° on a 12-hour light/dark cycle and fed with *Artemia* two or three times a week. DNA for genome sequencing were isolated from about 1000 polyps that were clonally cultured. Before genomic DNA extraction, symbiotic algae in *H. viridissima* were removed by photobleaching with 5 μM DCMU (3-(3,4-dichlorophenyl)-1,1-dimethylurea), as described previously (Pardy 1976; Habetha *et al.* 2003). To remove contamination from other organisms, polyps were starved and treated with antibiotics (50 mg/L ampicillin, rifampicin, neomycin, and streptomycin) for one week.

After several rounds of washing in sterilized culture medium, polyps were lysed in DNA extraction buffer (10 mM Tris-HCl, pH 8.0, 100 mM NaCl, 25 mM EDTA, pH 8.0, 0.5% SDS) and digested with 100 mg/L Proteinase K. Genomic DNA was extracted using the standard phenol-chloroform method with 100 mg/L RNaseA treatment. The quantity of DNA was determined using a NanoDrop (Thermo Fisher Scientific, Waltham, MA, USA), and the quality of high molecular-weight DNA was checked using agarose gel electrophoresis.

### Sequencing of genomic DNA

In paired-end library preparations for genome sequencing, genomic DNA was fragmented with a Focused-ultrasonicator M220 (Covaris Inc., Woburn, MA, USA). A paired-end library (average insert size: 540 bp) and mate-pair libraries (average insert sizes: 3.2, 4.6, 7.8 and 15.2 kb) were prepared using Illumina TruSeq DNA LT Sample Prep Kits and Nextera Mate Pair Sample Preparation Kits (Illumina Inc., San Diego, CA, USA), following the manufacturers’ protocols. These libraries were quantified by Real-Time PCR (Applied Biosystems StepOnePlus, Thermo Fisher Scientific) and quality checked using capillary electrophoresis on a Bioanalyzer. Genome sequencing was performed using the Illumina Miseq system with 600-cycle chemistry (2 × 300 bp). Genome sequencing statistics is shown in Table S1A.

### RNA extraction and sequencing

Total RNA was extracted from about 1000 polyps in six different conditions (with or without symbiotic algae, in light or dark conditions, and treated with antibiotics or DMCU with symbiotic algae) using Trizol reagent (Thermo Fisher Scientific) and an RNeasy Mini kit (QIAGEN, Hilden, Germany). The quantity of RNA was determined with a NanoDrop (Thermo Fisher Scientific). Quality of total RNA was checked with a BioAnalyzer (Agilent Technologies, Santa Clara, CA, USA). For mRNA-seq, libraries were produced using an Illumina TruSeq Stranded mRNA Sample Prep Kit and were sequenced on HiSeq 2000 instruments using 2 × 150-cycle chemistry. mRNA-sequencing statistics are shown in Table S1B.

### Assembly and gene prediction

Sequencing reads of genomic DNA were assembled using the Newbler Assembler, version 2.8 (Roche, Penzberg, Germany), and subsequent scaffolding was performed with SSPACE (Boetzer *et al.* 2011). Gaps inside scaffolds were closed with paired-end and mate-pair data using GapCloser of the Short Oligonucleotide Analysis Package (Luo *et al.* 2012). Then one round of Haplomerger2 processing pipeline (Huang *et al.* 2017) was applied to eliminate redundancy in scaffolds and to merge haplotypes. For gene model prediction, we used a species-specific gene prediction model that was trained based on mapping of the *Hydra viridissima* transcriptome and raw RNAseq reads against the genome assembly. Mapping and gene structure annotation were performed using the PASA pipeline v2.01 and were used to train AUGUSTUS software (Haas *et al.* 2003; Stanke *et al.* 2006). Genome completeness was evaluated using BUSCO (Benchmarking Universal Single-Copy Ortholog) (Seppey *et al.* 2019). RNA-Seq transcripts were mapped to the genome assembly with BWA.

### Genome size estimation

Genome size was estimated from raw paired-end reads by k-mer distribution analysis. Jellyfish v2.0.0 was used to count k-mers and their frequencies (Marçais and Kingsford 2011). The *Hydra viridissima* genome size was estimated from k-mer distribution frequencies using the GenomeScope web tool (Vurture *et al.* 2017) (http://qb.cshl.edu/genomescope/).

### Analysis of repetitive elements

Repetitive elements in the draft genome assembly of *Hydra viridissima* were identified *de novo* with RepeatScout version 1.0.5 (http://www.repeatmasker.org/RepeatModeler) and RepeatMasker version 4.0.6 (http://www.repeatmasker.org). Repetitive elements were filtered by length and occurrence so that only sequences longer than 50 bp and present more than 10 times in the genome were retained. The resulting sets of repetitive elements were annotated using BLASTN and BLASTX searches against RepeatMasker.lib (35,996 nucleotide sequences) and RepeatPeps.lib (10,544 peptides) bundled with RepeatMasker version 4.0.6. The results of both searches were combined, and BLASTX results were given priority in cases where BLASTN and BLASTX searches gave conflicting results.

### Analysis of Hydra viridissima genes

For comparative analysis of *H. viridissima* genes among cnidarians, protein sequences were obtained from Hydra 2.0 web portal (https://research.nhgri.nih.gov/hydra/) and the Compagen server (http://www.compagen.org) for *Hydra vulgaris* (*H. magnipapillata*) strain 105, from JGI (https://genome.jgi.doe.gov/Nemve1/Nemve1.home.html) for *Nematostella vectensis* (Putnam *et al.* 2007), from MARIMBA (available at http://marimba.obs-vlfr.fr/organism/Clytia/hemisphaerica) for *Clytia hemisphaerica* (Leclère *et al.* 2019), from the genome project website of OIST Marine Genomics Unit (https://marinegenomics.oist.jp/gallery/gallery/index) for *Acropora digitifera* (Shinzato *et al.* 2011), for *Morbakka virulenta* and for the Atlantic Ocean strain of *Aurelia aurita* (Khalturin *et al.* 2019). We used protein models derived from the Hydra 2.0 assembly of the *H. vulgaris* genome for all comparative analyses with *H. viridissima* as this assembly has higher continuity (scaffold N50 ∼1Mbp) and BUSCO values than the originally published assembly (Chapman *et al.*, 2010). For comparative reasons, statistics and results obtained with the Hydra 1.0 assembly (Chapman *et al.*, 2010) and Hydra 2.0 assembly (https://research.nhgri.nih.gov/hydra/) are shown side by side in Table 1 and Tables 4-7.

**Table 1:** Comparison of the genome assembly statistics of cridarians

	Hydrozoa				Scyphozoa	Cubozoa	Anthozoa	
Species	*Hydra viridissima**^a^*	*Hydra vulgaris (Hydra magnipapillata)*		*Clytia hemisphaerica*^b^	*Aurelia aurita*^c^	*Morbakka virulenta*^c^	*Acropora digitifera*^d^	*Nematostella vectensis*^e^
Geographical origin	Laboratory strain A99	Laboratory strain 105		Villefranche (Atlantic)	Baltic Sea (Atlantic)	Seto Inland Sea (Pacific)	Okinawa (Pacific)	Laboratory strain
		*ver. 1*^f^	*ver. 2*^g^					
Genome size (Mbp)	284	852	854	445	377	952	420	457
Number of Scaffolds	2,677	20,914	5,525	7,644	2,710	4,538	2,420	10,804
Longest scaffold (Mbp)	5.1	0.91	4.4	2.9	4.4	14.5	2.5	3.3
Scaffold N50 (Mbp)	1.1	0.1	1.0	0.4	1.0	2.2	0.5	0.5
GC content (%)	24.7	25.4	25.4	35	34.7	31.4	39	39
Repeats (%)	37.5	57	60.3	41	44.7	37.4	12.9	26
Gap rate (%)	16.8	7.82	8.0	16.6	6.63	11.9	15.2	16.6
Number of genes	21,476	31,452	33,820	26,727	28,625	24,278	23,668	27,273
Mean gene length (bp)	7,637	6,873	12,378	5,848	10,215	21,444	8,727	4,500
Mean exon length (bp)	209	247	N/A	281	368	350	316	208
Mean intron length (bp)	838	N/A	N/A	N/A	1391	3572	1057	800
BUSCO (complete) %	83.9	76.8	80.2	86.4	79.8	81.5	74.5	91.4

a This study. ^b^ Leclère *et al.*, 2019, ^c^Khalturin *et al.*, 2019. ^d^Shinzato *et al.*, 2011. ^e^Putnam *et al.*, 2007, ^f^Genome assembly version 1, Chapman *et al.*,2010, ^g^Genome assembly version 2, Hydra2.0 Web Portal (https://research.nhgri.nih.gov/hydra/).

In comparative analyses, domain searches against the Pfam database (Pfam-A.hmm) were performed using HMMER (Finn *et al.* 2016), and ortholog gene grouping employed OrthoFinder (Emms and Kelly 2015). To classify homeodomain-containing proteins, BLAST searches and phylogenetic analyses were performed. Homeodomain sequences in various animals were obtained from the Homeobox Database (http://homeodb.zoo.ox.ac.uk/families.get?og=All) (Zhong and Holland 2011).

For phylogenetic analysis, multiple alignments were produced with CLUSTALX (2.1) with gap trimming (Larkin *et al.* 2007). Sequences of poor quality that did not align well were deleted using BioEdit (Hall 1999). Phylogenetic analyses were performed using the Neighbor-Joining method (Saitou and Nei 1987) in CLUSTALX with default parameters (1,000 bootstrap tests and 111 seeds). Representative phylogenetic trees were drawn using FigTree v1.4.4 (http://tree.bio.ed.ac.uk/software/figtree/). Gene/protein IDs used for phylogenetic analysis are shown in the trees (Figs S2 and S3).

### Data availability

This whole-genome shotgun sequencing project has been deposited at DDBJ/ENA/GenBank under BioSample ID SAMN09635813 and BioProject ID: PRJNA480404. RNA-seq reads have been deposited at SRA of NCBI (SRX6792700-SRX6792705). Genome sequences, gene models, and a genome browser are also accessible at the website of the OIST Marine Genomics Unit Genome Project (https://marinegenomics.oist.jp/hydraviridissima_A99). A genome browser was established for assembled sequences using the JBrowse 1.12.3 (Skinner *et al.* 2009). Gene annotations from the protein domain search and BLAST search are likewise shown on the site. Reagents, software and datasets used in this study are listed in the Reagent Table. k-mer frequency distribution plots in the *Hydra viridissima* A99 genome is found in Figure S1. Phylogenetic analysis of ANTP genes is in Figure S2. Phylogenetic analysis of PRD genes is presented in Figure S3. Sequencing statistics for *Hydra viridissima* A99 are in Table S1. A summary of repetitive sequences in the *Hydra viridissima* A99 genome assembly are found in Table S2. Pfam domain-containing genes in the *Hydra viridissima* A99 genome are available in Table S3. Orthologs enriched in *Hydra viridissima* A99 (A) and *Hydra* (B) are in Table S4. Gene IDs of ANTP genes in *Hydra viridissima* A99 are in Table S5. Supplemental material available at figshare: https://doi.org/10.25387/g3.12911426.

## Results and Discussion

### Genome architecture of Hydra viridissima

*Hydra viridissima* appears green because of the symbiotic *Chlorella* that inhabit endodermal epithelial cells, and it is smaller than the brown hydra, *Hydra vulgaris* (Figure 1C). We decoded the genome of *H. viridissima* strain A99, which is closely related to strain A14c, Wuerzburg and J8 (Figure 1B). We previously reported the genome of its specific symbiotic alga, *Chlorella* sp. A99, and demonstrated that metabolic co-dependency exists between *H. viridissima* A99 and the symbiont (Hamada *et al.* 2018).

The genome of *H. viridissima* A99 was sequenced using the Illumina platform with paired-end and mate pair libraries. Statistics of sequence reads, the assembly, and genome architecture are shown in Table 1. We obtained ∼7,070 Mbp of paired-end sequences, and 4,765, 4,769, 3,669 and 3,551 Mbp for 3.2k, 4.6k, 7.8k, and 15.2k insert-size pate-pair sequences, respectively, comprising a total of ∼23,826 Mbp (Table S1). The size of the *H. viridissima* genome was estimated at ∼254 Mbp using k-mer analysis (k-mer = 19) based on paired-end sequence data (Fig. S1). This indicates that we achieved more than 90-fold sequence coverage of the genome. On the other hand, the total length of the genome sequence assembly reached 284,265,305 bp. That is, the total assembly closely matched the estimated genome size.

Although genomic DNA was extracted from a clonally propagated culture of hydra polyps maintained in the laboratory, heterozygosity was comparatively high (2.28% of the entire sequence) (Fig. S1). Thus, polyps originally collected from the wild had a high level of heterozygosity. Repetitive sequences constituted 37.5% of the genome and the gap rate was 16.8% of the genome (Table 1; see next section). Scaffolds from the present analysis numbered 2,677 and the scaffold N50 was 1.1 Mbp, with the longest scaffold reaching 5.1 Mbp (Table 1). The GC content of the genome was 24.7% (Table 1), suggesting that *H. viridissima* has an AT-rich genome similar to that of *H. vulgaris* (25.4%). Using 67,339,858,036 nucleotides of RNA-sequence data (Table S1), we predicted gene models. The genome was estimated to contain 21,476 protein-coding genes (Table 1). We did not find any gene models with sequence similarities to the symbiotic *Chlorella*. The mean gene length, exon length, and intron length were 7,637 bp, 209 bp, and 838 bp, respectively (Table 1). Compared to *H. vulgaris*, the green hydra has a compact genome, with 36.5% fewer genes (Table 1). The BUSCO value for the *H. viridissima* assembly is 84% for complete gene models and with inclusion of partial sequences, the genome accounts for 91% of the metazoan reference gene set (Table 1). Comparison of *H. viridissima* genome statistics with those of other cnidarian genomes showed that the *H. viridissima* genome assembly is comparable or of even better quality in regard to the scaffold N50 and BUSCO completeness (Table 1).

During assembly and gene annotation, we noticed that scaffold2223, composed of 18,375 base pairs (bp), contained almost the entire *H. viridissima* mitochondrial genome. The mitochondrial genome of *H. viridissima* strain A99 was linear, as reported by Bridge *et al.* (1992) and Pan *et al.* (2014b) for the other green hydras, while in brown hydra, *Hydra vulgaris*, mitochondrial genome is composed of two linear molecules (Bridge *et al.* 1992; Pan *et al.* 2014a; Pan *et al.* 2014b).

### Repetitive sequences in the Hydra viridissima genome

**Figure 2:**
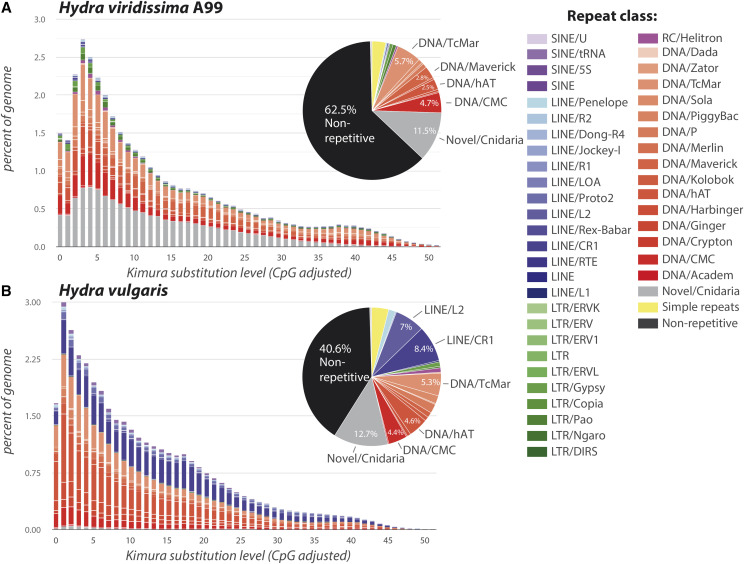
Interspersed Repeat Landscape in *Hydra*. Components and proportions of repetitive sequences in the genome of (A) *Hydra viridissima* A99 and (B) *H. vulgaris* are shown. Classes of repeat are shown in the right column.

Although the abundance of repetitive sequences in anthozoan genomes is generally low (15∼17%), genomes of medusozoans and hydrozoans have comparatively high levels of repetitive sequences, 60% in *H. vulgaris*, 41% in *Clytia*, 45% in *Aurelia*, and 37% in *Morbakka* (Table 1). This was also true of *H. viridissima* (37.5%) (Table 1). DNA transposons were the most abundant type, accounting for approximately 22.41% of the genome (Figure 2A, Table S2). Of these, TcMariner, CMC, Maverick and hAT were the largest components (Figure 2A). On the other hand, percentages of LTR retrotransposons (1.63%) and non-LTR retrotransposons (0.99%) were comparatively low (Figure 2A, Table S2).

In comparing repetitive elements between *H. viridissima* and *H. vulgaris*, it became apparent that DNA transposons (DNA/TcMar, DNA/hAT and DNA/CMC) occupy a similar portion of both *Hydra* genomes (Figure 2). In addition, novel and potentially cnidarian-specific repetitive elements occupy ∼12% of both genomes. Second, long, interspersed nuclear elements LINE/L2 (∼7%) and LINE/CR1 (8.4%) are large components of the *H. vulgaris* genome, but they are almost absent in the *H. viridissima* genome. It was suggested that a burst of retrotransposons occurred in the brown hydra lineage after divergence from the green hydra lineage, and may account for the large genomes of brown hydras (Chapman *et al.* 2010; Wong *et al.* 2019). Because *H. viridissima* occupies a basal position in the *Hydra* lineage, and the genome of another hydrozoan, *Clytia*, is smaller (∼450Mbp) and has fewer repetitive elements (41%) than *H. vulgaris*, the ancestral *Hydra* genome was likely rather compact, with fewer retrotransposons. Molecular and evolutionary mechanisms involved in the insertion of LINE components in the *H. vulgaris* genome will be a subject of future studies in relation to diversification and speciation within the *Hydra* clade.

### Innate immunity-related protein genes in the Hydra viridissima genome

Using the Pfam-domain search method, we surveyed genes for protein domains in the *H. viridissima* genome. We found approximately 4,500 different Pfam domains in this species (Table S3), a number comparable to those of other cnidarians. To identify the domains that are enriched in the *H. viridissima* genome, we counted the number of genes with each Pfam domain in cnidarian genomes, and selected the domains of which number are ≥2x higher in the green hydra genome than those of non-symbiotic cnidarians and show significant difference based on Chi-Square test (p-value < 0.001) (Table 2A). Then we checked the number of *H. viridissima*-enriched domains in the genome of the coral, *Acropora digitifera*, since it is also a symbiotic cnidarian. NACHT and NB-ARC, which have similar structures and functions, were the two most highly enriched domains and TIR occurred in the top 10 (Table 2A). NACHT/NB-ARC and TIR domains are found in the pattern-recognition receptors, Nod-like and Toll-like, which are sensors for pathogen- and damage-associated molecules. It appears that these domains are also enriched in *Acropora digitifera*, but not in non-symbiotic cnidarians (Table 2A).

**Table 2:** Number of genes with Pfam domains enriched in the *Hydra viridissima* genome and comparison of their number in the other cnidarian genomes. A. Domains enriched in *Hydra viridissima*. B. Domains enriched in *Hydra* species

A. Domain	*Hvir*	*Hvul*	*Ch*	*Aa*	*Mv*	*Nv*	*Ad*	Chi test*
**NACHT**	161	75	42	23	45	39	458	1E-53
**NB-ARC**	106	28	18	4	20	6	220	5E-53
**ATPase_2**	64	19	28	13	14	19	36	7E-35
**TIR_2**	49	11	19	15	24	17	49	7E-16
**DUF4218**	47	13	16	0	12	4	5	2E-66
**Endonuclease_7**	46	9	3	5	1	1	0	1E-189
**RAG1**	42	13	1	0	4	1	0	2E-160
**TIR**	41	7	3	11	15	12	36	1E-22
**CbiA**	23	7	6	6	7	6	2	8E-19
**MarR_2**	21	6	4	0	0	4	3	6E-40
**HTH_Tnp_IS630**	15	5	1	1	1	1	0	4E-40
**DUF2961**	14	5	2	3	3	1	3	5E-15
**TMEM151**	10	4	2	0	3	3	6	4E-06
**DUF1294**	5	1	1	1	2	0	0	6E-07

B. Domain	*Hvir*	*Hvul*	*Ch*	*Aa*	*Mv*	*Nv*	*Ad*	Chi test*
**DDE_3**	365	481	16	31	16	6	17	0E+00
**Dimer_Tnp_hAT**	296	376	11	44	52	21	27	0E+00
**HTH_Tnp_Tc3_2**	266	268	15	23	13	0	3	0E+00
**HTH_32**	185	176	14	25	4	4	9	1E-307
**ANAPC3**	182	157	61	37	36	46	44	2E-117
**HTH_23**	166	200	6	33	15	8	20	1E-225
**zf-BED**	116	116	14	8	9	19	5	1E-156
**HTH_29**	95	121	7	19	1	2	5	8E-148
**HTH_psq**	93	165	3	3	6	1	1	3E-189
**HTH_28**	85	87	2	6	5	6	11	5E-130
**SRP_TPR_like**	83	87	6	2	1	1	1	2E-163
**DUF4806**	69	54	6	0	1	0	0	3E-157
**PAX**	61	57	3	23	4	9	8	4E-63
**BTAD**	32	33	7	1	3	3	3	4E-39
**Sigma70_r4_2**	28	31	2	0	2	1	3	3E-44
**HTH_Tnp_Tc3_1**	26	13	1	0	0	0	0	3E-87
**HTH_7**	21	14	2	0	4	1	1	5E-33
**CD225**	21	19	9	2	4	7	7	4E-11
**DUF2738**	21	12	1	0	0	1	0	3E-56
**Sigma70_r4**	17	16	1	1	1	0	2	2E-26
**IGFBP**	15	21	7	5	4	1	3	1E-09
**Sulfate_transp**	15	18	2	4	5	4	5	3E-08
**DUF1280**	13	31	4	0	4	3	2	3E-17
**HTH_3**	13	11	1	5	2	2	1	4E-11
**Polysacc_deac_1**	13	14	3	6	1	4	2	5E-08
**DUF4817**	12	9	2	0	1	0	0	6E-21
**Ca_chan_IQ**	12	16	5	4	5	2	3	1E-05
**Torsin**	11	11	2	2	2	5	5	2E-05
**CIDE-N**	10	7	1	2	3	2	1	1E-07
**GRDP-like**	9	7	2	2	1	2	3	2E-05
**Transposase_mut**	8	10	1	1	3	0	0	2E-08

*Hvir: Hydra viridissima A99, Hvul: Hydra vulgaris (Hydra2.0), Ch: Clytia hemisphaerica, Mv: Morbakka virulenta, Aa: Aurelia aurita, Nv, Nematostella vectensis, Ad: Acropora digitifera,* *Chi-test: evalue of Chi-square test.

Expansion of genes for NACHT-containing proteins and TIR-containing proteins is supported by identification of orthologous protein groups by OrthoFinder. Genes for proteins similar to Nod-like receptor were the most overrepresented group and TIR-only proteins occurred in the top 7 in the *H. viridissima* genome (Table 3A, Table S4). However, these orthologs were not scored in the *Acropora* genome, suggesting that these proteins expanded in *H. viridissima* are different from those expanded in *Acropora*.

**Table 3:** Top 10 overrepresented orthologs in the *Hydra viridissima* genome and comparison of their gene number in the other cnidarian genomes. A. Orthologs enriched in the *Hydra viridissima* genome. B Orthologs enriched in *Hydra* species

A. Ortholog ID	*Hvir*	*Hvul*	*Ch*	*Aa*	*Mv*	*Nv*	*Ad*	Chi-test*	Annotation
**OG0000023**	152	51	30	19	0	0	0	8E-128	Nod-like receptor like
**OG0000049**	98	48	11	4	15	26	1	2E-58	Uncharacterized protein
**OG0000100**	73	29	0	0	0	1	0	3E-79	Uncharacterized protein
**OG0000191**	29	13	11	0	0	8	0	3E-17	Uncharacterized protein
**OG0000159**	24	4	0	8	2	0	0	7E-21	Uncharacterized protein
**OG0000602**	23	6	2	0	0	0	0	3E-24	Uncharacterized protein
**OG0000766**	23	1	1	0	0	0	1	2E-30	TIR-only protein
**OG0000525**	19	8	1	0	0	0	0	2E-18	Uncharacterized protein
**OG0001051**	18	1	0	0	0	0	0	1E-25	Uncharacterized protein
**OG0000975**	14	6	2	0	0	0	1	1E-11	DDE superfamily endonuclease

B. Ortholog ID	*Hvir*	*Hvul*	*Ch*	*Aa*	*Mv*	*Nv*	*Ad*	Chi-test*	Annotation
**OG0000006**	299	339	28	21	29	3	7	2E-262	HTH domain containing transposase
**OG0000018**	204	130	9	14	0	1	0	5E-194	ATP-dependent DNA helicase PIF1-like
**OG0000015**	185	128	46	14	1	0	45	6E-122	TPR containing
**OG0000008**	184	360	15	9	5	1	3	2E-249	DDE superfamily endonuclease
**OG0000027**	166	144	2	4	2	3	2	8E-163	Uncharacterized protein
**OG0000036**	95	154	1	1	7	1	1	4E-114	zinc finger domain containing transposase
**OG0000010**	82	352	12	38	19	11	2	4E-201	Uncharacterized protein
**OG0000073**	77	39	19	1	0	0	0	2E-66	Uncharacterized protein
**OG0000100**	73	29	0	0	0	1	0	5E-81	Uncharacterized protein
**OG0000093**	61	44	0	1	8	2	0	1E-52	Uncharacterized protein

*Hvir: Hydra viridissima A99, Hvul: Hydra vulgaris (Hydra2.0), Ch: Clytia hemisphaerica, Mv: Morbakka virulenta, Aa: Aurelia aurita, Nv, Nematostella vectensis, Ad: Acropora digitifera*, *Chi-test: evalue of Chi-square test.

**Figure 3:**
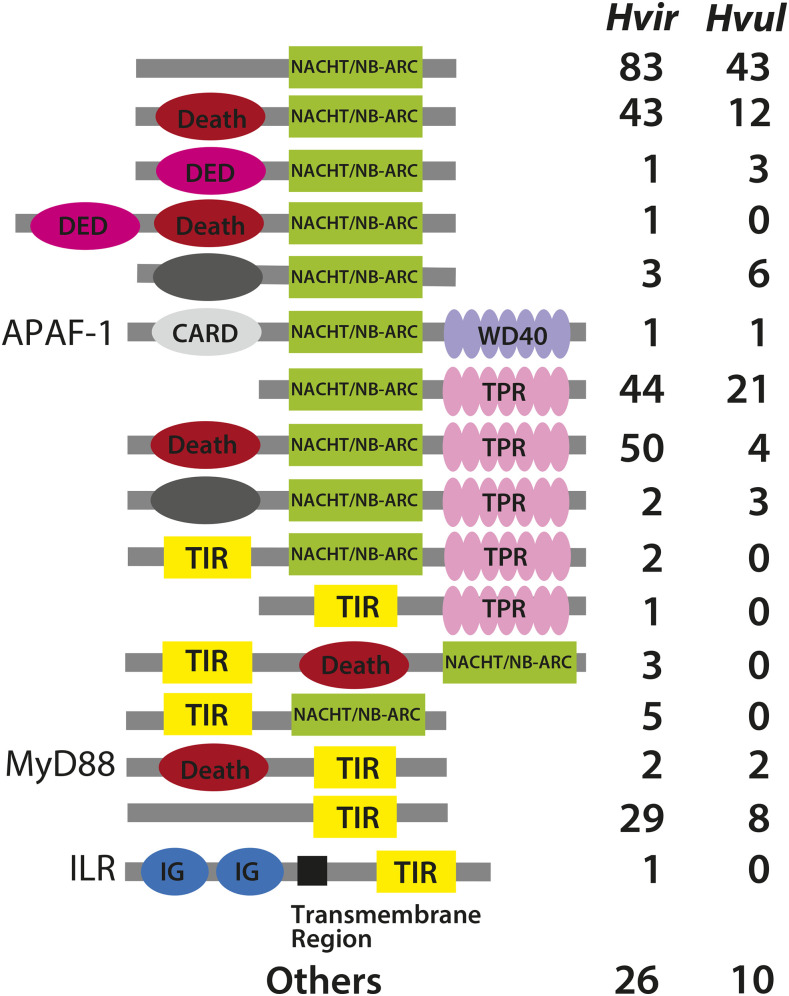
Schematic representation of domain structures of NACHT/NB-ARC or TIR-domain-containing proteins identified in *Hydra*. The domain structures and the number of NACHT/NB-ARC or TIR-domain-containing proteins in *Hydra viridissima* A99 (*Hvir*) and *H. vulgaris* (*Hvul*) are shown.

In *Acropora*, we previously showed unique, complex domain structures of proteins with NACHT/NB-ARC domains (Hamada *et al.* 2013). Thus, we further examined domain combinations of NACHT/NB-ARC proteins in *H. viridissima* to determine whether such complex domain structures are also found in this taxon. Basically Nod-like receptors have tripartite domain structures, consisting of effector-binding domains constituted of apoptosis-related domains, such as Death or DED in the N-terminus, NACHT/NB-ARC in the center, and a repeat domain that recognizes pathogen- and damage-associated molecules at the C-terminus. Humans have approximately 20 Nod-like receptor family proteins, and their ligand recognition region is a leucine-rich repeat (LRR). On the other hand, in Nod-like receptors of basal metazoans, not only LRR, but also tetratricopeptide repeats (TPR), WD40 repeats, and ankyrin repeats (Ank) are found as repeat domains. We previously showed that *Acropora* has all 4 types of Nod-like receptors, and that those with LRR are the most common (Table 4) (Hamada *et al.* 2013). In other cnidarians examined, only TPR and WD40 are found as repeat domains of Nod-like receptors, suggesting loss of the other types. Especially in *H. viridissima*, a larger number of genes for Nod-like receptors with TPR were found. In addition, their domain structures in *H. viridissima* vary widely, compared to those of *H. vulgaris* (Figure 3). For example, the domain combination of NACHT/NB-ARC with TIR, was found in *H. viridissima*, but not in *H. vulgaris*. In addition to NACHT-containing proteins, *H. viridissima* has more genes for TIR domain-containing proteins such as an interleukin-1 receptor (ILR), which are not found in *H. vulgaris*.

**Table 4:** The number of NACHT/NB-ARC domain-containing proteins in cnidarians and the combination of repeat domains

	*Hvir*	*Hvul* v1	*Hvul* v2	*Ch*	*Aa*	*Mv*	*Nv*	*Ad*
**Total**	264	89	101	56	58	24	6	489
**TPR**	103	38	29	3	8	4	2	57
**WD40**	2	4	2	3	2	3	1	8
**LRR**	0	0	0	0	0	0	0	125
**Ank**	0	0	0	0	0	0	0	11

Hvir: Hydra viridissima A99, Hvul v1: Hydra vulgaris (Chapman *et al.*, *2010**),Hvul v2: Hydra vulgaris (Hydra2.0), Ch: Clytia hemisphaerica, Mv: Morbakka virulenta, Aa: Aurelia aurita, Nv, Nematostella vectensis, Ad: Acropora digitifera,*

As mentioned above, diverse pattern-recognition receptor-related genes are found in both *H. viridissima* and *Acropora digitifera*. Their most significant shared attribute is symbiosis, the former with *Chlorella* and the latter with the dinoflagellate, *Symbiodinium*. Therefore, it is likely that the evolutionary development of symbiosis by certain cnidarians required expansion and greater sophistication of innate immunity genes. They may participate in recognition and maintenance of symbiotic organisms in cnidarian tissues. On the other hand, the structures (*e.g.*, repeat combination) of the Nod-like receptors most abundant in green hydras and corals are different. This indicates that species-specific adaptations to the environment and particular symbionts occurred independently in these lineages.

### Genes enriched in the genus Hydra

We further examined Pfam domains overrepresented specifically in *H. viridissima* and others present in both *H. viridissima* and *H. vulgaris*. This was done using the same criteria as above, that is, that the number of domains is ≥2x higher than those in other cnidarians and that the difference is significant by Chi-Square test (p-value < 0.001). (Table 2B).

Pfam domain searches and ortholog protein grouping demonstrated that *H. viridissima and H. vulgaris* possess many genes encoding domains that function in DNA binding. For example, genes containing transposase-related domain (DDE_3, Dimer_Tnp_hAT and Transposase_mut) and DNA-binding motif (HTH: helix-turn-helix, zf: zinc finger, Sigma70_r4, CIDE-N, RAG1) were overrepresented in both *H. viridissima* and *H. vulgaris* (Table 2B). In addition, ortholog protein grouping suggested that genes for HTH domain-containing transposase, ATP-dependent DNA helicase PIF1-like protein, DDE superfamily endonuclease, and zinc finger domain-containing transposase were overrepresented in both *Hydra* species (Table 3A). Although the functions of these genes are unknown, they may be involved in genome structure maintenance of *Hydra*, which contains many transposable elements.

Pfam domain searches also demonstrated that genes for proteins containing Sulfate_transp domain and those containing Polysacc_deac_1 domain are enriched in both *H. viridissima* and *H. vulgaris* (Table 2B). Sulfate_transp is found in the sulfate permease family, which is involved in uptake or exchange of inorganic anions, such as sulfate. So far, their functions in *Hydra* are unknown, but may be related to their limnetic life styles, which require active ion uptake. Polysacc_deac_1 is found in polysaccharide deacetylase, including chitin deacetylase, which is involved in chitin metabolism. It may contribute to construction of the extracellular matrix surrounding the body or structure of nematocytes, or molecular recognition events such as immune responses to pathogens with chitinous cell wall (Balasubramanian *et al.* 2012; Elieh Ali Komi *et al.* 2018; Rodrigues *et al.* 2016).

### Gene families for transcription factors and signaling molecules

Using Pfam-supported families, we examined the number of gene families for putative transcription regulator genes and signaling molecules (Table 5), since these genes are essential in development and physiology of metazoans. While major signaling pathways are present in cnidarians, some specialization in Cnidaria is known. For example, Wnt genes, which are important for oral-aboral body axis formation, diversified in the cnidarian lineage (Kusserow *et al.* 2005; Khalturin *et al.* 2019). Table 5A shows numbers of putative transcription factor genes in the *H. viridissima* genome. Zinc finger proteins (C2H2 type) were most abundant, with 105 members, although the abundance of this family has been noted in other cnidarian genomes (Khalturin *et al.* 2019). There were 33 HLH domain-containing and 50 homeobox domain-containing genes (Homeodomain-containing genes of *H. viridissima* are discussed in the next section). A similar analysis of putative signaling molecule genes showed that the *H. viridissima* genome contains 16 fibroblast growth factor (FGF)-like domain genes, 11 transforming growth factor-beta (TGB-β) genes, and 10 Wnt genes (Table 5B). These numbers are comparable to those in *H. vulgaris*. In general, the number of transcription factor and signaling molecule family members appeared similar among cnidarians, although a few families, such as AT_hook and Hairly-orange of transcription factors (Table 5A) and Interleukin 3 (IL3) families of signaling molecules (Table 5B) were not found in *Hydra* genomes.

**Table 5:** Number of putative transcription factor genes (A) and signaling molecule genes (B) in the *Hydra viridissima* genome.

A. Domain	*Hvir*	*Hvul v1*	*Hvul v2*	*Ch*	*Aa*	*Mv*	*Nv*	*Ad*
**ARID**	8	7	9	10	10	8	5	8
**AT_hook**	0	0	0	1	2	0	0	0
**bZIP_1**	26	26	30	26	22	25	36	29
**bZIP_2**	25	22	23	31	24	27	32	17
**CUT**	1	0	1	3	1	1	2	1
**DM**	6	6	5	7	9	11	12	7
**Ets**	9	11	11	13	21	14	16	12
**Forkhead**	17	17	16	19	15	26	34	22
**GATA**	4	4	5	3	7	7	4	5
**Hairy_orange**	0	0	0	1	4	2	6	7
**HLH**	33	36	34	44	52	50	72	53
**HMG_box**	30	33	33	31	30	29	33	27
**Homeobox**	50	44	49	70	88	82	153	96
**Hormone_recep**	9	9	9	12	12	9	20	9
**P53**	2	1	3	2	1	2	3	3
**PAX**	61	23	57	3	4	23	9	8
**Pou**	2	3	2	3	4	4	6	4
**RHD_DNA_bind**	2	1	3	5	2	2	3	2
**SRF-TF**	2	2	2	4	3	2	3	1
**T-box**	6	7	7	11	10	9	14	10
**TF_AP-2**	1	1	1	2	3	2	1	2
**zf-C2H2**	105	123	121	244	233	118	169	90
**zf-C2HC**	1	2	2	3	2	3	3	4
**zf-C4**	9	8	8	11	8	9	19	12

B. Domain	*Hvir*	*Hvul v1*	*Hvul v2*	*Ch*	*Aa*	*Mv*	*Nv*	*Ad*
**Cbl_N**	1	1	1	2	0	1	0	1
**Cbl_N2**	1	1	1	1	0	1	0	1
**Cbl_N3**	1	1	1	2	2	1	0	1
**DIX**	1	1	3	3	3	3	4	2
**FGF**	16	12	16	10	18	13	13	13
**Focal_AT**	1	2	3	1	1	1	0	0
**G-alpha**	29	28	27	29	29	32	37	22
**G-gamma**	2	2	3	1	7	5	4	3
**IL3**	0	0	0	1	0	0	0	0
**PDGF**	1	2	0	5	2	3	1	6
**Phe_ZIP**	1	0	1	1	1	1	0	1
**Rabaptin**	1	2	1	1	1	1	1	1
**RGS**	13	13	12	14	16	16	13	11
**RGS-like**	2	3	2	1	2	1	0	1
**STAT_alpha**	1	0	0	1	1	2	2	1
**STAT_bind**	2	1	3	1	1	1	1	1
**STAT_int**	2	1	1	1	0	1	0	1
**TGF_beta**	11	11	11	7	9	9	7	10
**TGFb_propeptide**	8	10	9	4	6	8	6	9
**wnt**	10	13	11	12	15	17	26	15

Hvir: Hydra viridissima A99, Hvul v1: Hydra vulgaris (Chapman *et al.*, *2010**),Hvul v2: Hydra vulgaris (Hydra2.0), Ch: Clytia hemisphaerica, Mv: Morbakka virulenta, Aa: Aurelia aurita, Nv, Nematostella vectensis, Ad: Acropora digitifera.*

### Hox and Para-Hox genes in Hydra viridissima

Among transcription factors, homeodomain-containing proteins have been intensively investigated as key molecules in the developmental toolkit. They are highly diversified and participate in a wide variety of developmental processes in metazoans. In particular, those in Cnidarians that are shared by the common ancestors of deuterostomes and protostomes are important to understand body plan evolution of bilaterians (Ferrier and Holland 2001; Chourrout *et al.* 2006; Ferrier 2016; DuBuc *et al.* 2018). While many orthologous genes of known homeodomain-containing proteins, including Hox and ParaHox genes, have been identified in cnidarians, cnidarian-specific specializations, such as loss of some homeodomain protein genes and fragmentation of the Hox cluster have been reported (Kamm *et al.* 2006; Steele *et al.* 2011; Chapman *et al.* 2010; Leclère *et al.* 2019). To understand the evolutionary trajectory of homeobox protein genes in the *Hydra* lineage, we classified them into ANTP (HOXL and NKL), PRD, LIM, POU, PROS, SINE, TALE, CERS, or ZF using bi-directional BLAST searches against sequences of homeodomains in other animals, using HomeoDB (Zhong and Holland 2011) (Table 6, Table S5) and phylogenetic analysis for ANPT- and PRD-class genes (Figs. S2 and S3), referring to the Hox genes previously identified in other cnidarians (Schummer *et al.* 1992; Chourrout *et al.* 2006; Leclère *et al.* 2019; Khalturin *et al.* 2019).

**Table 6:** Number of genes for the subclass of homeodomain-containing proteins in cnidarians

Class	Medusozoa						Anthozoad	
	Hydrozoa									
	*Hvir*	*Hvul* v1	*Hvul* v2	*Ch*	*Mv*	*Aa*	*Nv*	*Ad*
ANTP-HOXL	5	7	7	6	13	13	17	9
**ANTP-NKL**	9	8	11	20	21	20	65	33
**PRD**	21	16	16	18	25	28	43	31
**LIM**	4	4	5	5	5	5	5	4
**TALE**	4	3	4	5	4	10	6	5
**SINE**	2	2	2	4	5	4	6	4
**POU**	2	2	2	3	4	4	6	4
**CERS**	1	2	1	1	1	1	1	1
**CUT**	0	0	0	0	0	0	1	2
**HNF**	0	0	0	0	1	0	1	1
**PROS**	0	0	0	0	0	0	0	0
**ZF**	0	0	0	0	0	0	0	0
**Total**	48	44	48	62	79	85	151	94

Hvir: Hydra viridissima A99, Hvul v1: Hydra vulgaris (Chapman *et al.*, *2010**), Hvul v2: Hydra vulgaris (Hydra2.0), Ch: Clytia hemisphaerica, Mv: Morbakka virulenta, Aa: Aurelia aurita, Nv, Nematostella vectensis, Ad: Acropora digitifera.*

In the *H. viridissima* genome, we identified 48 homeodomain-containing genes in the genome, 5 ANTP-HOXL, 9 ANTP-NKL, 21 PRD, 4 LIM, 4 TALE, 2 SINE, 2 POU, and 1 CERS; however, we failed to find CUT, HNF, PROS and ZF classes. This tendency toward gene loss is shared by the two other hydrozoans, *H. vulgaris* and *Clytia hemishpaerica* (Table 6). Among cnidarians, anthozoan genomes (*Nematostella* and *Acropora*) apparently contain the most homeodomain-containing genes, while scyphozoans (*Aurelia*) and cubozoans (*Morbakka*) have intermediate numbers, and hydrozoan genomes contain the fewest. CUT class genes are not found in medusozoan genomes and all cnidarian genomes lack PROS and ZF class genes altogether. In addition, NKL genes are less abundant in *Hydra* and HOXL genes are less abundant in hydrozoans generally, than in other cnidarians.

**Figure 4:**
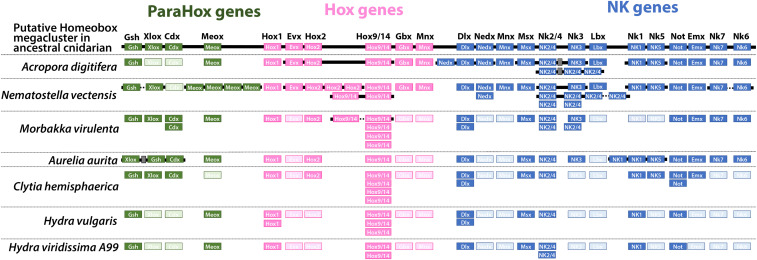
ParaHox, Hox and NK genes in cnidarians. The putative Homeobox megacluster in the last common ancestor of cnidarians (top) and homeobox genes and their cluster structures in extant cnidarians are represented. ParaHox genes (green boxes); Hox genes (pink boxes); NK genes (blue boxes). Empty boxes indicate lost genes. Horizontal lines (black) indicate chromosome fragments.

*H.viridissima* and *H. vulgaris* possess the same ANTP genes (Figure 4, Table 7, Table S5), suggesting a reason for the same body plan in these *Hydra* species, although the body size of *H. viridissima* is smaller. As previously reported (Leclère *et al.* 2019; Khalturin *et al.* 2019; Gauchat *et al.* 2000; Quiquand *et al.* 2009), ParaHox genes *Gsh* and *Meox* are present in *Hydra*, whereas *Xlox* and *Cdx* are missing, unlike other medusozoans (Table 7, Figure 4). On the other hand, Hox gene composition is quite similar among medusozoans. They have *Hox1 and Hox9-14*, but lack *Hox2*, *Evx*, *Gbx*, *Mnx*, unlike anthozoans. Medusozoans have lost many NKL genes, *Nedx*, *Hlx*, *Mnx*, *Msx*, and *Lbx* compared to anthozoans. In addition, *Dbx*, *Hlx*, *Nk3*, *Nk6* and *Nk7* are not found in hydrozoans, nor are *Nk5*, *Exm* or *Msxlx* in *Hydra* (Table 7, Figure 4). In addition, some degree of synteny conservation of HOXL genes and NKL genes is found in Anthozoa, but not in Medusozoa (Figure 4), suggesting complete fragmentation of the homeobox gene cluster in the common ancestor of medusozoans. *Nematostella* expresses *Gbx*, *Hlx*, *Nk3* and *Nk6* in the pharyngeal or mesenteric region (*Gbx* in pharyngeal endoderm (Matus *et al.* 2006), *Hlx* and *Nk6* in pharyngeal ectoderm, and *Nk3* in nutrient-storing somatic gonads in mesentery (Steinmetz *et al.* 2017)). Anthozoans have a pharynx and a mesentery that structurally supports the pharynx and serves as the site of gamete production in the gastrovascular cavity, while these tissues are not found in *Hydra*. Loss of these genes reflects the simplification of body structure in the *Hydra* lineage.

**Table 7:** Number of homeodomain-containing genes in the *Hydra viridissima* genome.

Class	Subclass	Familiy	*Hvir*	*Hvul v1*	*Hvul v2*	*Ch*	*Aa*	*Mv*	*Nv*	*Ad*
ANTP	**HOXL**	**Cdx**	0	0	0	1	2	1	0	0
		**Evx**	0	0	0	0	0	0	1	1
		**Gbx**	0	0	0	0	0	0	1	1
		**Gsx**	1	1	1	1	1	1	1	1
		**Hox1**	1	1	2	1	1	1	1	1
		**Hox2**	0	0	0	0	0	0	3	1
		**Hox9-13**	2	3	3	1	4	5	3	1
		**Meox**	1	1	1	0	1	1	4	1
		**Mnx**	0	0	0	0	0	0	1	1
		**Xlox/Pdx**	0	0	0	1	1	1	1	1
	**NKL**	**Barx**	1	1	1	0	1	1	4	2
		**Dbx**	0	0	0	0	1	1	2	1
		**Dlx**	1	2	2	2	1	2	1	1
		**Emx**	0	0	0	1	1	1	2	1
		**Hhex**	1	1	1	1	1	1	1	1
		**Hlx**	0	0	0	0	0	1	3	2
		**Lbx**	0	0	0	0	0	0	1	1
		**Msx**	1	1	1	1	0	0	1	1
		**Msxlx**	0	0	0	1	1	1	2	0
		**Nedx**	0	0	0	0	0	0	2	2
		**Nk1**	1	1	1	1	2	1	1	1
		**Nk2.1/2.2/4**	2	1	1	1	1	3	8	4
		**Nk3**	0	0	0	0	1	1	1	1
		**Nk5/Hmx**	0	0	0	1	1	0	1	1
		**Nk6**	0	0	0	0	1	1	1	1
		**Nk7**	0	0	0	0	1	1	1	1
		**Noto**	1	1	1	2	1	1	6	2
		**Ro**	0	0	0	0	0	0	1	0
		**Tlx**	0	0	0	1	1	1	0	0
PRD		**Alx**	0	0	0	0	1	1	1	1
		**Arx**	1	2	1	0	0	0	1	1
		**Dmbx**	1	0	1	1	1	0	7	2
		**Gsc**	1	1	1	1	1	1	1	1
		**Hbn**	1	1	1	1	2	1	1	1
		**Otp**	2	2	2	1	1	1	1	1
		**Otx**	3	3	2	3	4	7	3	4
		**Pax3/7**	0	0	0	0	0	0	2	2
		**Pax4/6**	2	1	1	2	2	1	2	1
		**Pitx**	1	1	1	1	1	1	1	1
		**Prox**	0	0	0	0	0	0	1	1
		**Rax**	0	0	0	0	1	1	1	1
		**Repo**	0	0	0	0	0	0	1	0
		**Uncx**	1	1	1	1	1	1	2	2
		**Vsx**	2	0	1	1	2	1	1	0
LIM		**Isl**	0	0	0	1	1	1	1	0
		**Lhx1/5**	1	0	1	1	1	1	1	1
		**Lhx2/9**	0	0	0	1	0	1	1	1
		**Lhx6/8**	1	1	1	1	1	1	1	1
		**Lmx**	1	1	1	1	1	1	1	1
POU		**Hdx**	0	0	0	1	0	0	0	0
		**Pou1**	0	0	0	0	1	1	1	0
		**Pou3**	0	0	0	0	1	1	3	2
		**Pou4**	1	1	1	1	1	1	1	1
		**Pou6**	1	1	1	1	1	1	1	1
SINE		**Six1/2**	0	0	0	1	2	2	2	2
		**Six3/6**	1	1	1	1	1	2	1	1
		**Six4/5**	1	1	1	1	1	1	2	1
TALE		**Irx**	1	1	1	1	2	1	1	1
		**Meis**	1	1	1	1	5	1	1	1
		**Pbx**	1	1	2	1	1	1	1	1
		**Pknox**	1	0	0	1	1	1	1	1
		**Tgif**	0	0	0	0	0	0	1	1
CERS		**Cers**	2	0	1	2	1	1	1	1
CUT		**Onecut**	0	0	0	0	0	0	1	1
HNF		**Hnf1**	0	0	0	0	0	1	0	1

Hvir: Hydra viridissima A99, Hvul v1: Hydra vulgaris (Chapman *et al.*, *2010**), Hvul v2: Hydra vulgaris (Hydra2.0), Ch: Clytia hemisphaerica, Mv: Morbakka virulenta, Aa: Aurelia aurita, Nv, Nematostella vectensis, Ad: Acropora digitifera.*

## Conclusions

In this study, we report the first genome assembly of *H. viridissima*, which is one the most basal species in the genus *Hydra* and the only species with symbiotic algae. Compared to *H. vulgaris*, *H. viridissima* has a compact genome one-third the size and with 36.5% fewer genes (Table 1). In addition, the *H. viridissima* genome has fewer repetitive sequences. RNA transposons, in particular, are almost absent (Figure 2). On the other hand, the repertoire of transcription factor genes, including homeodomain-containing genes in *H. viridissima* is quite similar to that in *H. vulgaris* (Table 5), reflecting the common body plan in these species. Comparative analysis of homeodomain genes among cnidarians indicates gradual simplification of the ANTP gene repertoire in the *Hydra* lineage (Tables 6 and 7, Figure 4), which is likely to reflect the simple body structure of *Hydra* and the absence of jellyfish and planula stages. In addition, we found diverse innate immunity genes in the *H. viridissima* genome that are also observed in corals (Table 4, Figure 3), indicating a common feature involved in algal symbiosis. The *H. viridissima* genome presented here provides a *Hydra* genome comparable in quality to those of other cnidarians, including medusozoans and anthozoans, which will hopefully facilitate further studies of cnidarian genes, genomes, and genetics to understand basal metazoan evolution and strategies to support algal symbiosis in cnidarians.
